# The Impact of Superficial Vessel Density on Glaucoma Progression according to the Stage of Glaucoma

**DOI:** 10.3390/jcm10215150

**Published:** 2021-11-02

**Authors:** Jiyun Lee, Chan Kee Park, Hae-Young Lopilly Park

**Affiliations:** Department of Ophthalmology, Seoul St. Mary’s Hospital, College of Medicine, The Catholic University of Korea, Seoul 06591, Korea; lemonssung@gmail.com (J.L.); ckpark@catholic.ac.kr (C.K.P.)

**Keywords:** optical coherence tomography angiography, advanced glaucoma, superficial vessel density, visual field progression

## Abstract

Purpose: To investigate the clinical significance of vessel density (VD) on visual field (VF) progression regarding the severity of glaucoma. Methods: A total of 130 eyes were recruited in this retrospective and longitudinal study. Superficial and deep VDs in circumpapillary and macular regions were measured via ImageJ. The rate of VF progression was defined as the mean deviation (MD) slope (dB/year). Linear regression was used to verify factors affecting deterioration of VF. The eyes with lower superficial VD were further analyzed. Results: Fifty patients with early glaucoma (EG) (MD > −6 dB) and 52 patients with moderate-to-advanced glaucoma (MAG) (MD ≤ −6 dB) were included. A faster progression rate was found in MAG (*p* = 0.049). Superficial VD was noticeably related to the VF progression rate in total eyes and in MAG (Both Ps ≤ 0.007, respectively). With patients in the lower half of the superficial VD, the VD was significantly associated with the rate of progression (B, 0.049, *p* = 0.021). This association was independent of the baseline MD and OCT parameters. Conclusion: Decreased superficial VD might conversely affect the progression of glaucoma even in MAG, which suggests superficial VD could be used as a potential marker to foresee the disease progression even in progressed eyes.

## 1. Introduction

Glaucoma, a progressive optic neuropathy due to irreversible structural damages in optic nerve head (ONH), has been a leading cause of blindness worldwide [1]. Thus, efforts have been made to find a valuable parameter of predicting further worsening of glaucoma. In line with these efforts, optical coherence tomography (OCT) and standard automated perimetry (SAP) have played essential roles as standard tools for detecting and monitoring glaucoma [2,3,4,5,6]. Nonetheless, in advanced glaucoma, OCT has been considered incapable of and inadequate for tracking structural changes in ONH due to the floor effect [7,8,9,10]. Compared to OCT, SAP might seem to be a better choice, as the severity of the disease escalates. However, issues like poor test–retest variability in advanced stage yield inexact and incorrect test results [11,12]. Recently, optical coherence tomography angiography (OTCA) proved its competency as a diagnostic tool [13,14], and its better association with VF indices has been ascertained in eyes with advanced glaucoma compared to OCT [15,16].

VD loss detected by OCTA could occur as a result of RGC or axonal loss, and therefore, it is not surprising to see its correlation with the severity of disease. However, remaining VD and blood flow could impact the status of the remaining RGC or axons. Additionally, we assumed that this influence would be different according to the stage of glaucoma, since advanced stage of glaucoma may possess higher proportion of dysfunctional RGC that could be vulnerable to blood flow. According to precedent studies [17,18], the forte of OCTA in evaluating glaucoma progression by assessing VD has been broadly and generally reported and investigated. In this study, unlike previous reports, we investigated the clinical significance of VD on VF progression by laser-focusing on their relationship according to glaucomatous stage, and by further analyzing the relationship between eyes with baseline VD in the upper and lower half and its impact on VF progression.

## 2. Materials and Methods

This study was conducted using a retrospective, longitudinal design and followed all relevant tenets of the Declaration of Helsinki. The Institutional Review and Ethics Boards (IRB) of Seoul St. Mary’s Hospital, South Korea approved this study. (KC21RISI0206), and informed consent was waived due to the characteristics of retrospective study’s design. 

### 2.1. Patients

We collected patient data from January 2010 to December 2020 using the electronic medical record, and those patients were referred to the glaucoma clinic at Seoul St. Mary’s Hospital for glaucoma screening. A total of 130 eyes were recruited for this study. Each participant underwent a comprehensive ophthalmic assessment, including the measurement of best-corrected visual acuity (BCVA), refraction, slit-lamp biomicroscopy, gonioscopy, Goldmann applanation tonometry, central corneal thickness (CCT) using ultrasound pachymetry (Tomey Corporation, Nagoya, Japan), the determination of axial length (AL) using ocular biometry (IOL Master; Carl Zeiss Meditec, Dublin, CA, USA), dilated stereoscopic examination of the optic disc and fundus, color disc photography, red-free RNFL photography (Canon, Tokyo, Japan), optical coherence tomography (Cirrus OCT using software version 6.0; Carl Zeiss Meditec) and Humphrey VF examination (24–2 Swedish Interactive Threshold Algorithm Standard program; Carl Zeiss Meditec), at initial work-up. After the initial visit, the follow-up was scheduled every 6 to 12 months according to patients’ disease severity. 

Regarding OCTA (DRI OCT Triton; Topcon), since the examination was available from 1 March 2016, the test was performed after that point.

Open-angle glaucoma diagnosis was defined by glaucomatous optic disc appearances (such as diffuse or localized rim thinning, a notch in the rim, or a cup-to-disc ratio higher than that of the other eye by >0.2); VF consistent with glaucoma (a cluster of ≥3 non-edge points on the pattern deviation plot with a probability of <5% of the normal population, with one of these points having a probability of <1%, a pattern standard deviation with *p* < 5%, or a Glaucoma Hemifield Test result outside the normal limits in a consistent pattern on two qualifying VFs), confirmed by two glaucoma specialists (H.Y.-L.P. and C.K.P.); and an open angle on gonioscopy. Early glaucoma (EG) was defined as mean deviation (MD) of VF more than −6 dB, and moderate to advanced glaucoma (MAG) was defined as MD −6 dB or less [19].

The inclusion criteria were: follow-up for a minimum of 5 years with at least 5 reliable VF tests excluding the initial test, a BCVA of ≥20/40, intraocular pressure (IOP) under 21 mmHg, OCT images with signal intensity of 6 or more and OCTA image quality scores greater than 50.

The exclusion criteria were as follows: poor OCT images (due to involuntary saccadic movement, misalignment, or artifacts, and signal strengths < 6); a history of any retinal disease, including diabetic or hypertensive retinopathy or other retinal complications; a history of eye trauma or surgery, including glaucoma incisional surgery or laser treatment; or a history of systemic or neurological diseases possible to affect the VF. If both eyes were eligible, one eye was randomly selected from each patient.

### 2.2. Optical Coherence Tomography Angiography

The microvasculature of the peripapillary and macular areas was imaged via swept-source OCTA device (DRI OCT Triton; Topcon), which uses a laser with a wavelength of 1050 nm and scan speed of 100,000 A scans per second. The OCTA provided en face images through automated layer segmentation around the optic nerve head into 4 layers. Among those, we chose the radial peripapillary capillary (RPC) mode, which estimate a 70-μm-thick layer below the internal limiting membrane (ILM), to assess the superficial layer. As for the deep layer, the original choroidal/disc mode was customized for the measurement. Namely, it was intended to measure from 130 μm below the ILM to 390 μm below Bruch’s membrane; however, we manually reset the starting point as retinal pigment epithelium (RPE) layer to exclude the changes of the superficial layer. As per macular, its superficial layer was defined as the region between 2.6 μm below the ILM to 15.6 μm below the junction of the inner plexiform layer (IPL) and INL. The macular deep layer corresponds to the region from 15.6 μm below IPL/INL to 70.2 μm below IPL/INL. 

To calculate VD, we used Image J software (National Institutes of Health, Bethesda, MD, USA), and the estimation was performed in the same manner as that of our previous group [20,21].

Images with quality score less than 50 and unclear ocular vascular structures were excluded for further analysis. In addition, the quality of images was independently evaluated by three clinicians (J.L., H.-Y.L.P. and C.K.P.). 

### 2.3. Assessment of Visual Field Progression

The rate of VF progression was calculated as the MD slope and recorded as decibels per year (dB/year). To increase the specificity and sensitivity, we included patients with at least 5 reliable VF tests and ruled out the initial result from the calculation. The presence of VF progression was evaluated with trend-based analysis using Guided Progression Analysis software. If the change slope was statistically significant (*p* < 0.05), a noticeable progression was considered. 

### 2.4. Statistical Analysis

All statistical analyses were performed using the SPSS statistical package (SPSS, Inc., Chicago, IL, USA), and student’s *t*-tests were adopted to compare the differences between the groups. The chi-squared test or Fisher’s exact test was applied to compare frequencies. A *p*-value of less than 0.05 was considered statistically significant. Linear regression analysis was applied to explore meaningful factors affecting VF progression. Further analysis was carried out with patients experiencing VF progression. After categorizing these patients into two subgroups, one in the upper half of baseline superficial VD and the other in the lower half, student’s *t*-test and chi-square test or Fisher’s exact test were used for comparison. Further linear regression analysis was carried out to identify related factors with VF progression.

The variables with significance at *p* < 0.10 in univariate analysis were included in the multivariate model. *p* < 0.05 was considered to represent statistical significance.

## 3. Results

Despite 130 eyes recruited, 28 eyes were excluded; 23 (17.7%) eyes were ruled out due to poor OCTA image quality, and 5 (3.85%) eyes with low signal intensity of OCT images. The remaining 102 eyes from 50 patients with EG and 52 patients with MAG were included in the further analysis. Among 102 eyes, 65 (63.73%) eyes showed glaucoma progression based on trend-based GPA.

Patient’s characteristics are in Table 1. The total follow-up period for all patients was 95.41 ± 35.72 months. Substantial differences were found in OCT parameters with the exception of disc areas at both baseline and finial visits (All Ps ≤ 0.005) (Table 2). Unlike deep VDs, both superficial VDs had noticeable decreases in eyes with MAG, compared to EG (Both Ps <0.001) (Table 2).

**Figure 1:**
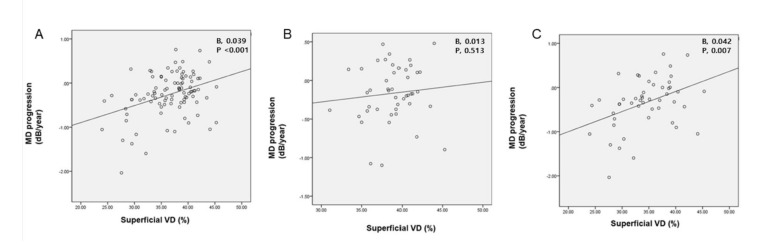
Relationship between circumpapillary superficial vessel density and visual field progression. A significant association between circumpapillary superficial vessel density (VD) and mean deviation (MD) progression was found in all eyes (*n*, 102) (**A**). In early stage (*n*, 50) (**B**), no significant association was found, while in moderate to advanced glaucoma (*n*, 52) (**C**), the VD had a substantial association. Linear regression analysis was used.

To identify factors associated with the rate of MD progression, linear regression analysis was adopted using MD slope as the dependent variable (Table 3). Since OCT parameters have been known for their robust associations with VF indices [22,23,24], we created two multivariate models: one with OCT parameters (model 1) and the other without OCT parameters (model 2). According to model 1, average RNFLT was the ultimate factor associated with the rate of MD progression. However, in model 2, we found that superficial VD was the sole significant factor associated with MD slope of the VF (β, 0.039, *p* < 0.001, Figure 1A).

The sub-analysis with EG eyes (Table 4) showed baseline IOP as the most crucial parameter associated with VF progression in both models. Unlike total eyes, there was no significant correlation found between circumpapillary superficial VD and MD progression (Figure 1B). In eyes with MAG, the result was quite incompatible with that of eyes with EG (Table 5). The analysis in multivariate model 1 showed that average RNFLT was the strongest associated factor to the rate of MD progression (β, 0.027, *p*, 0.001), but model 2 revealed circumpapillary superficial VD to be the strongest associated factor to the MD progression rate (β, 0.042, *p*, 0.007, Figure 1C) without the OCT parameters.

Although circumpapillary superficial VD was found to be a significant variable related to the rate of MD progression, it may be strongly related to the RFNLT. Thus, to elucidate the relationship between circumpapillary superficial VD and the rate of MD progression, further analysis was carried out with patients who presented VF progression based on trend-based GPA. A total of 65 eyes was found to show VF progression, and subsequently, we classified the patients into two sub-groups: 35 eyes in the lower half of baseline superficial VD and 30 eyes in the upper half of baseline superficial VD. 

No noticeable difference in characteristics between the two groups was found (Table S1 in Supplementary Materials). In Table 6, the rate of MD progression was markedly faster in eyes in the lower half of baseline superficial VD, compared to the upper half of baseline superficial VD (−0.58 ± 0.45, −0.38 ± 0.30, *p*, 0.041). Regarding VDs in OCTA (Table 7), superficial VDs in both circumpapillary and macular areas were significantly reduced in eyes with lower half of baseline superficial VD (Both Ps < 0.001), whereas no significant differences were found in either circumpapillary or macular deep VDs.

Linear regression analysis was performed in groups classified according to their VD levels to ascertain variables associated with the rate of MD progression. In eyes in the upper half of baseline superficial VD, no significant factor was found to be associated with VF progression. Instead, in eyes with VD in the lower half (Table 8), in multivariate analysis model 1, circumpapillary superficial VD, macular deep VD and average cup to disc ratio remained significant (β, 0.047, *p*, 0.016, β, 0.058, *p*, 0.026, β, 2.451, *p*, 0.003, respectively); however, in model 2, it was circumpapillary superficial VD that had the most notable association with the rate of MD progression (β, 0.049, *p*, 0.021).

## 4. Discussion

In this study, we found that circumpapillary superficial VD was one of the factors consistently associated with VF progression in glaucomatous eyes, particularly in eyes with MAG. However, the inclusion of OCT parameters resulted in somewhat mixed-up outcomes. Statistically, less associated factors may fail to show significance when two parameters are strongly associated, and this also could be apparent between OCT and VD parameters. That is why we think VD parameters did not show significance when all variables were included in the analysis. However, when sub-analyses were performed with groups classified by the remnant superficial VD, the association between superficial VD and MD slope was significant even in the regression analysis, including the OCT parameters. Also, the association was significantly independent to baseline MD, although the eyes in the lower superficial VD groups tended to be in the advanced stage of glaucoma. These results could be interpreted that circumpapillary superficial VD is an important parameter associated with VF progression. This shows that the remaining VD is more important in eyes with VD loss or reduced VD.

According to the latest report, macular and circumpapillary VDs reached the estimated floor in a much more advanced stage, compared to RNFLT and GCIPLT [25]. Additionally, Shin et al. found that in eyes with MAG, circumpapillary VD showed noticeably more robust association to VF mean sensitivity than RNFLT [16,26]. Another study also reported a remarkable relationship between VD and the severity of VF damage was irrespective of the structural loss [27]. All these results support the notion of OCTA’s capability of surveilling glaucoma aggravation, and our study findings are among them. 

Nevertheless, in the analysis including OCT parameters, average RNFLT overpowered the impact of superficial circumpapillary VD in MAG eyes as well as all patients. Therefore, we extracted the patients with definite VF progression, and sub-analyzed the relationship among the parameters. The result showed that it was the circumpapillary superficial VD that had the significant association with MD progression in the eyes with lower half of baseline superficial VD, regardless of including OCT parameters. However, there was no significant factor found in the eyes with upper half of remnant superficial VD. These findings are in line with precedent histologic reports that there was merely moderate agreement between retinal ganglion cell (RGC) losses and RNFL thinning [28,29], indicating that even if a decrease in RNFLT exists, there could be fragile, weak but still functional RGCs. Our findings could be explained in this regard. Specifically, although lamina cribrosa has already been collapsed during the progression of the disease, the sustainable vessels feeding vulnerable RGCs would be operating till the death of the RGCs. Consequently, superficial circumpapillary VD could have a noticeable association with MD progression in the progressed glaucoma and be a better indicator of status of RGCs compared to structural changes.

In contrast to the strong relationship between superficial circumpapillary VD and MD progression in MAG, in EG, neither structural nor vascular loss were associated with MD progression. Instead, baseline IOP was the only significant factor. This finding is consistent with the “mechanical theory”, which proposed RGC death to result from high IOP [30]. Moreover, with respect to precedent studies, IOP has a lot to do with glaucoma development and progression in early stage of the disease [31,32,33]. However, unlike our findings, Moghimi et al. [34] saw that reduced VD in the macula and optic nerve head at baseline were associated with a faster RNFL thinning in mild to moderate glaucoma, while IOP was not. We presume the discrepancy between ours and their studies might be due to adoption of the respective parameter of evaluating glaucoma progression. Since circumpapillary superficial VD has a strong correlation with RNFLT [35,36,37], RNFL thinning could show a stronger relationship with VD, not IOP. 

In terms of association between macular deep VD and VF progression, previously, our group found that decreased macular deep VD was a crucial risk factor for aggravation of both general and central VF defects [20,21]. We found that macular deep VD was significantly associated with VF deterioration in eyes in the low superficial VD at baseline. However, the outcome was reasonable solely with the analysis including OCT parameters. In eyes with MAG, macular deep VD showed borderline association with VF progression. In the analysis without OCT parameters, circumpapillary superficial VD was the noticeable factor, whereas macular deep VD was not. The discrepancy between the two studies might stem from the different grouping; our precedent study analyzed patients as a whole group, but we classified patients by disease severity and amount of remnant VD. Therefore, when it comes to investigating and predicting VF progression in MAG, circumpapillary superficial VD appears to be advantageous, in particular for eyes whose level of the remnant superficial VD is relatively scarce.

This study has several limitations. First, our sample sizes were modest, and this could mean other variables may not have been fully represented in the present study. However, the total follow-up period for all patients was more than seven years, and we believe this long-term period might be sufficient to compensate for the weakness of the sample size. Second, because of inevitable weaknesses in a retrospective study including selection bias and lack of clarification of causal relationship, a prospective and longitudinal study is essential. Third, the fluctuation accuracy of VF test was possible. Therefore, to minimize the interference of this weakness, we accessed both trend-based GPA and MD slope for evaluation of VF progression. Fourth, VD measured by OCTA is not a true measure, but a surrogate of blood flow. Namely, this measurement does not quantify the flow rate within the detected vessels. Thus, longitudinal study is necessary to discern the impact of VD over VF progression. Lastly, there are issues regarding OCTA imaging interpretation, especially with deep layer. Specifically, retinal vessel signals evident on en face, and this hinders researchers from examining deep layer precisely [38,39]. Nevertheless, recent studies have found that repeatability and reproducibility in measurement were good in superficial layer as well as in deep layer [40,41].

## 5. Conclusions

It is notable that circumpapillary superficial VD was substantially associated with VF progression, especially in MAG or in eyes with low remnant VD. In other words, our findings indicate that superficial circumpapillary VD could be a potent predictor for monitoring the disease progression, especially in eyes with MAG.

## Figures and Tables

**Table 1:** Patients’ demographics.

	Early (*n*, 50)	Moderate to Advanced (*n*, 52)	*p* Value
Age (years)	54.90 ± 13.93	59.15 ± 10.88	0.090 ^a^
Male (*n*, %)	18 (36.0%)	27 (51.9%)	0.105 ^b^
DM (Yes, %)	4 (8.0%)	3 (5.8%)	0.656 ^b^
HTN (Yes, %)	10 (20.0%)	8 (19.2%)	0.922 ^b^
Total Follow-up Time (months)	99.64 ± 38.30	91.35 ± 32.92	0.243 ^a^
Baseline IOP (mmHg)	15.72 ± 2.72	15.92 ± 3.85	0.760 ^a^
CCT (μm)	532.57 ± 28.24	528.31 ± 34.85	0.515 ^a^
Axial Length (mm)	25.39 ± 1.71	24.74 ± 4.26	0.369 ^a^
Baseline MD (dB)	−2.17 ± 1.99	−11.84 ± 3.83	**<0.001** ^a^
Baseline PSD (dB)	3.33 ± 2.19	10.86 ± 3.43	**<0.001** ^a^
Final MD (dB)	−3.26 ± 3.21	−14.30 ± 5.99	**<0.001** ^a^
Final PSD (dB)	4.85 ± 3.24	12.04 ± 2.91	**<0.001** ^a^
Rate of MD Progression (dB/year)	−0.16 ± 0.36	−0.35 ± 0.56	**0.049** ^a^
Baseline Rim Area	0.85 ± 0.16	0.63 ± 0.26	**<0.001** ^a^
Baseline Disc Area	1.95 ± 0.42	1.94 ± 0.49	0.892 ^a^
Baseline Average CD Ratio	0.73 ± 0.08	0.79 ± 0.09	**<0.001** ^a^
Baseline Average RNFLT (μm)	77.76 ± 9.68	65.02 ± 10.61	**<0.001** ^a^
Baseline Average GCIPLT (μm)	71.64 ± 7.35	63.80 ± 7.86	**<0.001** ^a^
Final Rim Area	0.83 ± 0.15	0.67 ± 0.22	**<0.001** ^a^
Final Disc Area	1.96 ± 0.48	1.92 ± 0.49	0.676 ^a^
Final Average CD ratio	0.74 ± 0.08	0.79 ± 0.10	**0.005** ^a^
Final Average RNFLT (μm)	73.02 ± 9.90	62.88 ± 10.09	**<0.001** ^a^
Final Average GCIPLT (μm)	68.68 ± 8.50	63.16 ± 7.44	**0.001** ^a^

*n* = number; DM = diabetes mellitus; HTN = hypertension; IOP = intraocular pressure; CCT = central corneal thickness; MD= mean deviation; dB = decibel; PSD = pattern standard deviation; CD = cup disc; RNFLT = retinal nerve fiber layer thickness; GCIPLT = ganglion cell inner plexiform layer thickness. Mean values are presented with standard deviations. Bold font indicates significant *p* values (*p* < 0.05). ^a^ Student’s *t*-test; ^b^ chi-squared test.

**Table 2:** Comparison between early and moderate to advanced groups in optical coherence tomography angiography.

	Early (*n*, 50)	Moderate to Advanced (*n*, 52)	*p* Value
OCTA SSI	67.31 ± 5.43	64.88 ± 7.19	0.129
Circumpapillary Superficial VD (%)	38.86 ± 2.83	34.39 ± 5.08	**<0.001**
Circumpapillary Deep VD (%)	52.15 ± 5.85	53.79 ± 4.91	0.143
Macular Superficial VD (%)	35.51 ± 2.23	33.04 ± 3.02	**<0.001**
Macular Deep VD (%)	39.42 ± 2.30	38.97 ± 2.23	0.312

*n* = number; OCTA = optical coherence tomography angiography; SSI = signal strength index; VD = vessel density. Mean values are presented with standard deviations Bold font indicates significant *p* values (*p* < 0.05). Student’s *t*-test.

**Table 3:** Linear regression analysis to determine the correlation between variables and the rate of MD progression in total eyes (*n*, 102).

	Univariate		Multivariate: Model 1		Multivariate: Model 2	
	B	*p* Value	B	*p* Value	B	*p* Value
Age (year)	−0.005	0.152				
Baseline IOP (mmHg)	−0.028	**0.050**	−0.027	0.104	−0.017	0.247
Baseline MD (dB)	0.022	**0.010**	−0.006	0.689	0.003	0.757
OCTA						
Circumpapillary Superficial VD (%)	0.039	**<0.001**	0.006	0.756	0.039	**<0.001**
Circumpapillary Deep VD (%)	−0.009	0.353				
Macular Superficial VD (%)	0.042	**0.011**	0.021	0.290	0.016	0.402
Macular Deep VD (%)	0.023	0.284				
Baseline OCT						
Rim Area	0.432	**0.028**	−0.211	0.436		
Average CD Ratio	−0.461	0.403				
Average RNFLT (μm)	0.015	**<0.001**	0.020	**<0.001**		
Average GCIPLT (μm)	0.017	**0.021**	−0.003	0.744		

Model 1: analysis including the parameters of the OCT; Model 2: analysis excluding the parameters of the OCT. IOP = intraocular pressure; MD = mean deviation; dB = decibel; OCTA = optical coherence tomography angiography; VD = vessel density; OCT = optical coherence tomography; CD = cup to disc; RNFLT = retinal nerve fiber layer thickness; GCIPLT = ganglion cell inner plexiform layer thickness. Only variables with a *p* value < 0.10 in the univariate analysis were included in the multivariate model. Bold font indicates significant *p* values (*p* < 0.05).

**Table 4:** Linear regression analysis to determine the correlation between variables and the rate of MD progression in early glaucoma (*n*, 50).

	Univariate		Multivariate: Model 1		Multivariate: Model 2	
	B	*p* Value	B	*p* Value	B	*p* Value
Age (year)	0.002	0.628				
Baseline IOP (mmHg)	−0.054	**0.003**	−0.054	**0.004**	−0.054	**0.003**
Baseline MD (dB)	0.012	0.659				
OCTA						
Circumpapillary Superficial VD (%)	0.013	0.513				
Circumpapillary Deep VD (%)	0.003	0.758				
Macular Superficial VD (%)	0.017	0.472				
Macular Deep VD (%)	−0.028	0.220				
Baseline OCT						
Rim Area	0.581	**0.070**	0.567	0.055		
Average CD Ratio	−0.027	0.968				
Average RNFLT (μm)	0.004	0.451				
Average GCIPLT (μm)	0.002	0.880				

Model 1: analysis including the parameters of the OCT; Model 2: analysis excluding the parameters of the OCT. IOP = intraocular pressure; MD = mean deviation; dB = decibel; OCTA = optical coherence tomography angiography; VD = vessel density; OCT = optical coherence tomography; CD = cup to disc; RNFLT = retinal nerve fiber layer thickness; GCIPLT = ganglion cell inner plexiform layer thickness. Only variables with a *p* value < 0.10 in the univariate analysis were included in the multivariate model. Bold font indicates significant *p* values (*p* < 0.05).

**Table 5:** Linear regression analysis to determine the correlation between variables and the rate of MD progression in moderate to advanced glaucoma (*n*, 52).

	Univariate		Multivariate: Model 1		Multivariate: Model 2	
	B	*p* Value	B	*p* Value	B	*p* Value
Age (year)	−0.014	**0.052**	−0.004	0.592	−0.009	0.198
Baseline IOP (mmHg)	−0.014	0.487				
Baseline MD (dB)	0.031	0.135				
OCTA						
Circumpapillary Superficial VD (%)	0.045	**0.004**	0.009	0.694	0.042	**0.007**
Circumpapillary Deep VD (%)	−0.019	0.272				
Macular Superficial VD (%)	0.043	0.100				
Macular Deep VD (%)	0.066	**0.060**	0.070	0.052	0.063	0.061
Baseline OCT						
Rim Area	0.238	0.433				
Average CD ratio	−0.169	0.855				
Average RNFLT (μm)	0.024	**0.001**	0.027	**0.001**		
Average GCIPLT (μm)	0.019	**0.093**	−0.004	0.729		

Model 1: analysis including the parameters of the OCT; Model 2: analysis excluding the parameters of the OCT. IOP = intraocular pressure; MD = mean deviation; dB = decibel; OCTA = optical coherence tomography angiography; VD = vessel density; OCT = optical coherence tomography; CD = cup to disc; RNFLT = retinal nerve fiber layer thickness; GCIPLT = ganglion cell inner plexiform layer thickness. Only variables with a *p* value < 0.10 in the univariate analysis were included in the multivariate model. Bold font indicates significant *p* values (*p* < 0.05).

**Table 6:** Comparison in eyes with MD progression in terms of superficial vessel density.

	Eyes with Superficial VD Lower 50% (*n*, 35)	Eyes with Superficial VD Upper 50% (*n*, 30)	*p* Value
Baseline MD (dB)	−10.78 ± 5.92	−4.38 ± 4.90	**<0.001** ^a^
Baseline PSD (dB)	8.91 ± 4.47	5.58 ± 4.52	**0.004** ^a^
Final MD (dB)	−14.93 ± 7.38	−6.61 ± 5.18	**<0.001** ^a^
Final PSD (dB)	10.52 ± 3.50	8.0 ± 4.95	**0.024** ^a^
Rate of MD Progression (dB/year)	−0.58 ± 0.45	−0.38 ± 0.30	**0.041** ^a^
Baseline Rim Area	0.58 ± 0.28	0.81 ± 0.18	**<0.001** ^a^
Baseline Disc Area	1.97 ± 0.43	1.90 ± 0.36	0.479 ^a^
Baseline Average CD Ratio	0.81 ± 0.08	0.74 ± 0.07	**<0.001** ^a^
Baseline Average RNFLT (μm)	62.26 ± 9.89	76.40 ± 9.53	**<0.001** ^a^
Baseline Average GCIPLT (μm)	61.69 ± 7.64	71.35 ± 8.07	**<0.001** ^a^
Final Rim Area	0.65 ± 0.26	0.77 ± 0.14	**0.031** ^a^
Final Disc Area	2.02 ± 0.48	1.86 ± 0.40	0.173 ^a^
Final Average CD Ratio	0.81 ± 0.09	0.76 ± 0.07	**0.010** ^a^
Final Average RNFLT (μm)	59.60 ± 8.19	71.90 ± 9.54	**<0.001** ^a^
Final Average GCIPLT (μm)	60.70 ± 7.72	67.93 ± 8.97	**0.001** ^a^

*n* = number; VD=vessel density; DM = diabetes mellitus; HTN = hypertension; IOP = intraocular pressure; CCT = central corneal thickness; MD = mean deviation; dB = decibel; PSD = pattern standard deviation; CD = cup to disc; RNFLT = retinal nerve fiber layer thickness; GCIPLT = ganglion cell inner plexiform layer thickness. Mean values are presented with standard deviations Bold font indicates significant *p* values (*p* < 0.05). ^a^ Student’s *t*-test; ^b^ chi-squared test.

**Table 7:** Comparison of optical coherence Tomography Angiography Parameters between eyes with MD progression in terms of superficial vessel density.

	Eyes with Superficial VD Lower 50% (*n*, 35)	Eyes with Superficial VD Upper 50% (*n*, 30)	*p* Value
OCTA SSI	61.82 ± 8.05	61.0 ± 8.18	0.814
Circumpapillary Superficial VD (%)	32.05 ± 3.64	40.30 ± 2.11	**<0.001**
Circumpapillary Deep VD (%)	54.08 ± 5.02	52.49 ± 5.20	0.218
Macular Superficial VD (%)	32.35 ± 2.73	35.18 ± 2.69	**<0.001**
Macular Deep VD (%)	39.51 ± 2.66	39.13 ± 2.32	0.546

*n* = number; VD = vessel density; OCTA = optical coherence tomography angiography; SSI = signal strength index. Mean values are presented with standard deviations Bold font indicates significant *p* values (*p* < 0.05). Student’s *t*-test.

**Table 8:** Linear regression analysis to determine the correlation between variables and the rate of MD progression in eyes with presenting visual field progression in lower 50% of baseline superficial VD (*n*, 35).

	Univariate		Multivariate: Model 1		Multivariate: Model 2	
	B	*p* Value	B	*p* Value	B	*p* Value
Age (year)	−0.003	0.658				
Baseline IOP (mmHg)	0.012	0.567				
Baseline MD (dB)	0.018	0.166				
OCTA						
Circumpapillary Superficial VD (%)	0.049	**0.021**	0.047	**0.016**	0.049	**0.021**
Circumpapillary Deep VD (%)	−0.003	0.832				
Macular superficial VD (%)	0.046	0.108				
Macular Deep VD (%)	0.066	**0.022**	0.058	**0.026**		
Baseline OCT						
Rim Area	−0.383	0.173				
Average CD Ratio	1.685	**0.068**	2.451	**0.003**		
Average RNFLT (μm)	0.009	0.253				
Average GCIPLT (μm)	0.009	0.440				

Model 1: analysis including parameters related to optical coherence tomography; Model 2: analysis excluding parameters related to optical coherence tomography. IOP = intraocular pressure; MD = mean deviation; dB = decibel; OCTA = optical coherence tomography angiography; VD = vessel density; OCT = optical coherence tomography; CD = cup to disc; RNFLT = retinal nerve fiber layer thickness; GCIPLT = ganglion cell inner plexiform layer thickness. Only variables with a *p* value < 0.10 in the univariate analysis were included in the multivariate model. Bold font indicates significant *p* values (*p* < 0.05).

## Data Availability

The data presented in this study are available on request from the corresponding author. The data are not publicly available due to limited access to electronic medical records of Seoul St. Mary’s Hospital.

## Supplementary Materials

The following are available online at https://www.mdpi.com/2077-0383/10/21/5150/s1, Table S1: Comparison of patient’s characteristics in eyes with MD progression according to Superficial Vessel Density.
