# Supplementary material for: The Impact of Superficial Vessel Density on Glaucoma Progression according to the Stage of Glaucoma
Source: J Clin Med. 2021 Nov 2;10(21):5150. doi: 10.3390/jcm10215150 (PMC8585109; doi:10.3390/jcm10215150)
Supplement: Supplementary file 1 [file jcm-10-05150-s001.zip › jcm-1427322 supplementary.pdf]

Supplementary Material

Table S1. Comparison of patient's characteristics in eyes with MD progression according to Superficial Vessel Density

	Eyes with Superficial VD Lower 50% (*n*, 35)	Eyes with Superficial VD Upper 50% (*n*, 30)	*p* Value
**Age, mean (SD), years**	59.94 (12.40)	55.30 (11.56)	0.126 *
**Male, No. (%)**	19 (54.3)	12 (40)	0.250 #
**DM, No. (%)**	1 (2.9)	4 (13.3)	0.114 #
**HTN, No. (%)**	5 (14.3)	7 (23.3)	0.349 #
**Baseline IOP, mean (SD), mmHg**	17.0 (3.91)	15.57 (2.90)	0.103 *
**CCT, mean (SD), μm**	527.26 (34.92)	530.79 (29.51)	0.675 *
**Axial Length, mean (SD), mm**	25.51 (1.96)	24.93 (1.95)	0.298 *

VD = vessel density; *n* = number; DM = diabetes mellitus; HTN = hypertension; IOP = intraocular pressure; CCT = central corneal thickness. Mean values are presented with standard deviations Bold font indicates significant *p* values ($p < 0.05$).* Student's *t*-test; # Chi-squared test.
